# Chlamydia and gonorrhoea in pregnant Batswana women: time to discard the syndromic approach?

**DOI:** 10.1186/1471-2334-7-27

**Published:** 2007-04-16

**Authors:** Maria Romoren, Johanne Sundby, Manonmany Velauthapillai, Mafizur Rahman, Elise Klouman, Per Hjortdahl

**Affiliations:** 1 Institute of General Practice and Community Medicine, Faculty of Medicine, University of Oslo, Box 1130 Blindern, N-0318 Oslo, Norway; 2 The National Health Laboratory, Ministry of Health, Gaborone, Botswana; 3 Sexual and Reproductive Health Associates, Gaborone, Botswana; 4 Norwegian Institute of Public Health, Oslo, Norway

## Abstract

**Background:**

Chlamydia and gonorrhoea are major causes of morbidity among women in developing countries. Both infections have been associated with pregnancy-related complications, and case detection and treatment in pregnancy is essential. In countries without laboratory support, the diagnosis and treatment of cervical infections is based on the syndromic approach. In this study we measured the prevalence of chlamydia and gonorrhoea among antenatal care attendees in Botswana. We evaluated the syndromic approach for the detection of cervical infections in pregnancy, and determined if risk scores could improve the diagnostic accuracy.

**Methods:**

In a cross-sectional study, 703 antenatal care attendees in Botswana were interviewed and examined, and specimens were collected for the identification of *C trachomatis*, *N gonorrhoeae *and other reproductive tract infections. Risk scores to identify attendees with cervical infections were computed based on identified risk factors, and their sensitivities, specificities, likelihood ratios and predictive values were calculated.

**Results:**

The prevalence of chlamydia was 8%, and gonorrhoea was found in 3% of the attendees. Symptoms and signs of vaginal discharge did not predict cervical infection, and a syndromic approach failed to identify infected women. Age (youth) risk factor most strongly associated with cervical infection. A risk score with only sociodemographic factors had likelihood ratios equivalent to risk scores which incorporated clinical signs and microscopy results. However, all the evaluated risk scores were of limited value in the diagnosis of chlamydia and gonorrhoea. A cut-off set at an acceptable sensitivity to avoid infected antenatal care attendees who remained untreated would inevitably lead to considerable over-treatment.

**Conclusion:**

Although in extensive use, the syndromic approach is unsuitable for diagnosing cervical infections in antenatal care attendees in Botswana. None of the evaluated risk scores can replace this management. Without diagnostic tests, there are no adequate management strategies for *C trachomatis *and *N gonorrhoeae *in pregnant women in Botswana, a situation which is likely to apply to other countries in sub-Saharan Africa. Screening for cervical infections in pregnant women is an essential public health measure, and rapid tests will hopefully be available in developing countries within a few years.

## Background

Sub-Saharan Africa has the highest prevalence of gonorrhoea and chlamydia worldwide. These two sexually transmitted infections (STIs) have a major impact on health, particularly in women and neonates [1]. A cervical infection with *Neisseria gonorrhoeae *or *Chlamydia trachomatis *can cause serious complications, such as ascending infections, infertility, cervical cancer, spontaneous abortion, premature delivery and low birth weight [2]. Epidemiological and biological studies have shown that ulcerative and non-ulcerative STIs can enhance HIV transmission [3,4].

It should be part of the mandate of the antenatal care programmes to diagnose and treat *C trachomatis *and *N gonorrhoeae*, due to their association with maternal, foetal and infant morbidity. In developing countries, diagnosis of cervical infections is limited to the syndromic approach, using the 'vaginal discharge syndrome' (VDS) algorithm. In the early 1990s, the World Health Organization developed syndromic management guidelines as a case management of symptomatic STI patients in countries without laboratory support [5]. Easily recognized symptoms and signs are combined using flowcharts, and patients are treated with two or more antibiotic regimens to cover the majority of, or the most serious, organisms responsible for producing a syndrome. The VDS algorithm for the management of vaginal and cervical infections is far from ideal, and for chlamydia or gonorrhea, this simplified approach is neither sensitive nor specific [2,5-8]. Simple, rapid tests for these infections have been requested for more than a decade [9,10], and the continued use of the syndromic approach in the management of cervicitis has been viewed as a temporary solution while awaiting the development of such tests [11].

The majority of women with a cervical infection are asymptomatic [12] and their infection will not be detected by the syndromic approach. As screening with specific diagnostic tests have been out of reach, risk scores based on sociodemographic risk factors, symptoms or signs of infection, urine sticks and microscopy have been explored as screening tools to identify asymptomatic infections among pregnant women. Studies from sub-Saharan countries have shown variable and unconvincing results [9,10,13-16].

It has become clear that vaginal discharge is poorly predictive of cervicitis, and in order to reduce over-treatment, the WHO recommends the incorporation of risk assessments in the syndromic management of cervical infections [5]. In Botswana, the syndromic approach has recently been revised, and lower abdominal pain was included as a second entry symptom in the VDS algorithm [17]. Treatment for *C trachomatis *and *N gonorrhoeae *is reserved for women who present with vaginal discharge or lower abdominal pain *and *have either a positive risk assessment (age less than 21 years or complaints of yellow discharge) or yellow discharge or cervical mucopus on examination. These new recommendations are based on a non-validated study of female STI patients [18]. Risk factors' association with infection is specific to the population group from which they are extracted [5,13], and a validation of the algorithm in the antenatal care is lacking.

There are two parallel strategies to manage reproductive tract infections (RTIs) in pregnancy in Botswana. In addition to the syndromic management of women with symptoms, all antenatal care attendees are clinically screened for RTIs. The antenatal care guidelines recommend a routine speculum examination at the first antenatal visit, to "exclude genital infections, abnormalities and pelvic tumours" [19]. It is not uncommon for abnormal vaginal discharge to be found in women who are not eliciting symptoms. Asymptomatic pregnant women with signs of vaginal discharge will also be provided with syndromic treatment. Clinical screening bypasses the original entry point of the syndromic algorithms: symptoms which lead to health-care seeking, and it is not within the WHO recommendations.

The aim of this study was to determine the prevalence of *C trachomatis *and *N gonorrhoeae *among antenatal care attendees in Botswana, and to assess the validity of the 'vaginal discharge syndrome and lower abdominal pain' algorithm in the diagnosis of cervicitis in pregnancy. We also evaluated the diagnostic accuracy of risk scores based on sociodemographic factors, specific symptoms or signs, or microscopy. The results from Botswana are used to discuss the management of cervicitis in pregnancy in sub-Saharan Africa.

## Methods

Participating in this study were 703 pregnant women who visited the 13 main facilities providing antenatal care in Gaborone, Botswana: 12 primary health clinics and one outpatient department. A proportionate sample of attendees was recruited from each location. This proportion corresponded to the percentage of all antenatal care attendees in Gaborone who visited that facility during the previous year. Facilities were visited one-by-one by a medical doctor between October 2000 and February 2001. In the majority of clinics, all attendees were included in the study. In the busiest clinics, only a sample of the attendees was included; the selection of attendees in these clinics was incidental. Approximately one out of every four antenatal care attendees in Gaborone was included in the study during the period of data collection. All participants gave written, informed consent. The only exclusion criterion was the use of antibiotics during the previous two weeks.

A structured interview and information from the patient-held antenatal record were used to obtain data on sociodemographic and behavioural factors, current RTI symptoms, and diagnosis and prescribed treatment for such conditions earlier in the pregnancy. All patients underwent a genital examination; appropriate specimens were collected; and abnormal signs from external and internal genitalia were recorded in detail.

### Laboratory analyses

Urine was checked on site with a dipstick; all other specimens were analysed at the National Health Laboratory in Gaborone. Cervical swabs were obtained for ligase chain reaction (LCR) amplification technology for detection of *C trachomatis *and *N gonorrhoeae*. The swabs were placed in LCx^® ^transport media, transported to the laboratory the same day, and stored at -20°C prior to batch processing. The LCx^® ^Assays (Abbott Laboratories, IL) were performed according to the manufacturer's instructions. A case of *C trachomatis *or *N gonorrhoeae *infection was defined as an individual with a positive LCR analysis.

A high vaginal swab for identification of *Trichomonas vaginalis *was placed in Stuart transport media. Before culturing, a wet-mount was made and examined for the presence of motile trichomonads by light microscopy, 100 × magnification. The swab was then agitated into a bottle of Diamond's modified medium. The bottles were incubated with indicators in Oxoid gaspack jars (3.4 litres, with anaerobic system BR 038B). Wet-mounts from the cultures were examined once a day for five days by light microscopy.

Gram-stained vaginal smears were scored for bacterial vaginosis according to Nugent's criteria [20]. Culture of *Candida *species was initiated by direct inoculation of a high vaginal swab on Saboraud plates on site, and Gram-stained smears and wet-mounts from high vaginal swabs were examined for budding yeast cells and pseudohyphae. A cervical smear was gram-stained to count polymorphonuclear leukocytes per high power field (PMN/HPF).

### Statistical analyses and risk scores

Data were analysed with the statistical package SPSS Version 11. We performed univariate analyses on all independent variables in our dataset which could be associated with cervical infection. The factors were within four categories: sociodemographic and behavioural factors, symptoms, clinical signs, and microscopy results. The dependent variable "cervicitis" was defined as infection with *C trachomatis *or *N gonorrhoeae*, or both. Factors which in the univariate analysis were associated with infection at a 0.2 level (p-value of odds ratio (OR)) were included in multiple logistic regression analyses. We performed multiple logistic regression analyses on sociodemographic factors, symptoms, clinical signs and laboratory results, separately and combined. Due to the high number of variables, the full analysis of different factors' independent association with cervicitis had to be performed in two steps. First, multiple logistic regression analyses for the four variable categories were performed separately. The analysis was then extended to combining the factors from the different categories which were significantly independently associated with cervical infection in the first step.

Three levels of risk scores were computed and retrospectively applied, and each of them were assessed for their usefulness as a diagnostic tool to manage *N gonorrhoeae *and *C trachomatis *infections in the antenatal care. The first risk score level consists of only sociodemographic factors. At the second risk score level we added findings from the gynaecological examination. At the third level, we also allowed the results from microscopy of vaginal and cervical smears. Microscopy can be done on site, and thus all three risk score levels – the sociodemographic, the clinical and the microscopy score – can theoretically be performed during an antenatal care visit. The weights for each factor used in the risk scores were based on correspondent multiple logistic regression analysis models. The log of the OR for the variables in the model multiplied by 10 and rounded to the nearest whole number were used as weights for the respective factors in the risk scores [9]. The factors included and their respective weights are shown for each risk score in Table 1.

**Table 1:** Three levels of risk scores: Variables included and their respective weights

**Variables included**		**Sociodemographic score**	**Clinical score**	**Microscopy score**
		Weight*	Weight	Weight
**Sociodemographic factors**				
Age	<20	21	24	24
	20–29	13	14	14
	30+	0	0	0
Education	Primary school	10	12	11
	Junior secondary school	6	7	6
	Senior secondary or higher	0	0	0
Length of relationship	1 year or less	5	-	-
	Between 1 and 2 years	2	-	-
	2 years and more	0	-	-
Marital status	Unmarried	12	-	-
	Married	0	-	-
**Clinical factors**				
Amount of discharge	Moderate/profuse	-	2	2
	Scarce	-	0	0
Thin/runny discharge	Yes	-	8	8
	No	-	0	0
Smelly discharge	Yes	-	5	4
	No	-	0	0
Cervix abnormal	Yes	-	3	2
	No	-	0	0
**Microscopy results**				
White blood cell count in cervical smear	3–4+	-	-	14
	2+	-	-	10
	1+	-	-	7
	No/few	-	-	0

Cut-off at 40% sensitivity		Score >32	Score >28	Score >36
Cut-off at 70% sensitivity		Score >30	Score >21	Score >29

*The log of the OR from the correspondent multiple logistic regression analysis for each of the variables, multiplied by 10 and rounded to the nearest whole number

Receiver Operating Characteristic (ROC) curves were used to compare the different risk scores. The ROC curve shows the sensitivity and specificity that correspond to each possible cut-off for the risk score. The validity of different diagnostic strategies (the existing STI management guidelines, diagnosis based on symptoms or signs alone, and diagnosis based on a risk score) was assessed by measuring sensitivity, specificity, positive and negative likelihood ratios (LR+ and LR-) and positive and negative predictive values (PPV and NPV). The LCR-based laboratory diagnosis of cervical infection was used as the reference standard. In the evaluation and comparison of diagnostic strategies, we present two cut-offs for the risk scores, taken at a sensitivity of minimum 0.40 and 0.70.

The study was approved by ethical committees in Botswana and in Norway.

## Results

Of the 703 women, 67 (10%) had laboratory-confirmed cervical infection: 51 (8%) were infected with *C trachomatis*, and 21 (3%) with *N gonorrhoea*. *T vaginalis *was identified in 131 (19%) women and bacterial vaginosis in 268 (38%) women. Candida species were identified by microscopy and/or culture in 416 (59%) of the women. A total of 561 (80%) of the antenatal care attendees had one or more of these five reproductive tract infections.

### Subjective symptoms and clinical signs

In spite of the high prevalence of cervical and vaginal infections in this study, few women confirmed symptoms when probed. Complaints of vaginal discharge were elicited from 119 (17%) of the women and lower abdominal pain from 58 (8%). Vaginal discharge was the most common clinical sign; candidalike discharge was found in 81 (12%) and other abnormal discharge was found in 227 (32%) of the women.

### Factors associated with *C trachomatis *and/or *N gonorrhoeae*

Demographic, behavioural, and obstetric factors; symptoms, signs, and simple laboratory tests; and their association with *C trachomatis *and/or *N gonorrhoeae *infection are shown in Table 2. Age was the strongest predictor of cervical infection. The prevalence of infection was highest among teenagers (22%, 95% confidence interval (CI): 13 to 32), whereas two-thirds of the infections were within the largest age group: women 20–29 years. None of the evaluated symptoms predicted cervical infection. Symptoms of vaginal discharge or lower abdominal pain were therefore not included in any of the risk scores. There were 65 (9%) women who reported believing that they had a genital illness, but the prevalence of cervicitis was not significantly higher in this group (OR = 1.6, 95% CI: 0.8 to 3.4). Of clinical findings, vaginal (excluding candida-like) discharge was significantly associated with increased prevalence of cervicitis (OR = 1.8, 95% CI: 1.1 to 3.0). Several discharge characteristics (thin, smelly, foamy and increased amounts) were also significantly associated with cervical infection. Two laboratory results were associated with cervicitis; white blood cells in the cervical smear, and infection with *T vaginalis*.

**Table 2:** Univariate analyses on risk factors for cervicitis (*C trachomatis *and/or *N gonorrhoeae*) among 703 antenatal care attendees in Gaborone, Botswana

	**No.**	**Women with cervicitis**		**Odds ratio**	**Confidence interval**	**p-value**
					
		*No*.	*(%)*		*(95%)*	
**Sociodemographic factors**						
Age groups						
< 20	76	17	(22)	9.1	3.4–24.1	0.000
20–29	432	44	(10)	3.6	1.5–8.5	0.004
30+	195	6	(3)	1		
Education						
Primary school or less	168	17	(10)	1		
Junior secondary school	310	36	(12)	1.2	0.6–2.2	0.620
Senior secondary or higher	225	14	(6)	0.6	0.3–1.2	0.160
Marital status						
Married	114	2	(2)	1		
Unmarried	589	65	(11)	6.9	1.7–28.7	0.008
Partners last 12 months						
One partner	671	64	(10)	1		
Two or more	32	3	(9)	1.0	0.3–3.3	0.976
Time in relationship						
One year or less	118	19	(16)	2.5	1.4–4.6	0.003
1 to 2 years	137	16	(12)	1.7	0.9–3.2	0.094
>2 years	448	32	(7)	1		
**Subjective symptoms**						
Vaginal discharge						
No	584	55	(9)	1		
Yes	119	12	(10)	1.1	0.6–2.1	0.822
Lower abdominal pain						
No	650	63	(10)	1		
Yes	53	4	(8)	0.8	0.3–2.2	0.610
Thinks she has an infection						
No	638	58	(9)	1		
Yes	65	9	(14)	1.6	0.8–3.4	0.217
**Clinical signs**						
Vaginal discharge						
Negative	476	37	(8)	1		
Positive	227	30	(11)	1.8	1.1–3.0	0.023
Candida-like discharge						
Negative	622	61	(10)	1		
Positive	81	5	(6)	0.6	0.2–1.5	0.279
Moderate or profuse discharge						
Negative	447	34	(8)	1		
Positive	256	33	(13)	1.8	1.1–3.0	0.023
Yellow discharge						
Negative	438	38	(9)	1		
Positive	265	29	(11)	1.3	0.8–2.2	0.322
Thin/runny discharge						
Negative	615	48	(8)	1		
Positive	88	19	(22)	3.3	1.8–5.9	0.000
Foamy discharge						
Negative	590			1		
Positive	113	19	(17)	2.3	1.3–4.1	0.005
Smelly discharge						
Negative	659	58	(9)	1		
Positive	44	9	(21)	2.7	1.2–5.8	0.014
Cervical bleeding/erythroplaquia						
Negative	520	45	(9)	1		
Positive	183	22	(12)	1.4	0.8–2.5	0.184
**Laboratory analyses**						
Urine stix (nitritis/leucocytes)						
Negative	609	59	(10)	1		
Positive	94	8	(9)	0.9	0.4–1.9	0.718
WBC in cervical smear						
None/few	124	5	(4)	1		
1+	267	22	(8)	2.1	0.8–5.8	0.135
2+	171	18	(11)	2.8	1.0–7.8	0.048
3–4+	140	22	(16)	4.4	1.6–12.1	0.004
Candida (microscopy or culture)						
Negative	287	26	(9)	1		
Positive	416	41	(10)	1.1	0.7–1.8	0.724
Trichomoniasis						
Negative	571	41	(7)	1		
Positive	132	26	(20)	3.2	1.9–5.4	0.000
Bacterial vaginosis (BV)						
Negative	435	40	(9)	1		
Positive	268	27	(10)	1.1	0.7–1.9	0.700

The factors which were independently associated with cervical infection are shown in Table 3.

**Table 3:** Univariate and multiple logistic regression analysis of risk factors for cervicitis (*C trachomatis *and/or *N gonorrhoeae*) among 703 antenatal care clients in Gaborone, Botswana

	**Total women**		**Women with cervicitis**		**Multiple logistic regression**	
	No.	(%)	No.	(%)	Odds ratio	p-value

Age groups						
<20	76	(11)	17	(22)	10.5 (3.59–30.72)	0.000
20–29	432	(62)	44	(10)	4.0 (1.60–10.10)	0.003
30+	195	(28)	6	(3)	1	
Education						
Primary school or less	168	(24)	17	(10)	1	
Junior secondary school	310	(44)	36	(12)	0.6 (0.31–1.23)	0.168
Senior secondary or higher	225	(32)	14	(6)	0.4 (0.16–0.80)	0.013
Thin/runny discharge						
No	615	(87)	48	(8)	1	
Yes	88	(13)	19	(22)	2.1 (1.06–4.24)	0.035
WBC in cervical smear						
None/few	124	(18)	5	(4)	1	
1+	267	(38)	22	(8)	2.0 (0.72–5.52)	0.188
2+	171	(24)	18	(11)	2.7 (0.92–7.61)	0.070
3–4+	140	(20)	22	(16)	3.7 (1.31–10.66)	0.014

### Syndromic management and screening for cervical infections

**Figure 1:**
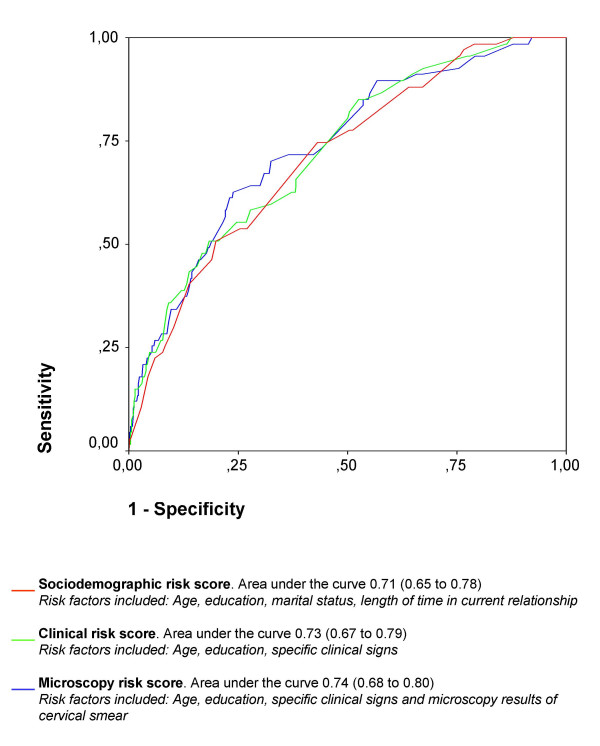
Receiver Operating Characteristics (ROC) curves for three levels of risk scores (sociodemographic, clinical and microscopy risk scores). The risk scores are based on multiple logistic regression analyses and used as a screening tool to identify *N gonorrhoeae *and *C trachomatis*. The risk scores are applied retrospectively on 703 antenatal care attendees in Gaborone, Botswana.

The sociodemographic, clinical, and microscopy-based risk scores performed similar in the management of *N gonorrhoeae *and *C trachomatis *infection, as illustrated with their ROC-curves (Figure 1). The diagnostic accuracy of the risk scores did not increase significantly when detailed information from the clinical examination and subsequently the microscopy results were added to the sociodemographic risk factors.

Table 4 presents the evaluated options for the diagnosis of *C trachomatis *and *N gonorrhoeae *in antenatal care in the absence of specific diagnostic tests: the syndromic algorithm, symptoms, signs, and the three risk scores. The VDS algorithm did not identify women with *C trachomatis *and *N gonorrhoeae *in our study population (positive likelihood ratio (LR+) 1.1, 95% CI: 0.6 to 1.9). Symptoms of vaginal discharge or lower abdominal pain in pregnancy proved inappropriate as an entry point to the VDS algorithm, with a LR+ of 0.94 (95% CI: 0.6–1.5). The current practice of clinical screening for signs of vaginal discharge (excluding candida-like discharge) also had low discriminative ability (LR+ 1.5, 95 % CI 1.1–1.9).

**Table 4:** Diagnostic strategies to identify infection with *Chlamydia trachomatis *and/or *Neisseria gonorrhoeae *in 703 antenatal care attendees in Botswana.

	**Positive on assessment**		**Cervical infection**		**Sensitivity**	**Specificity**	LR+*	**LR-**	**PPV**	**NPV**
	n	(%)	N	(%)						

**Symptoms and signs**										
VDS algorithm	104	(15)	11	(11)	0.16	0.85	1.12 (0.63–1.92)	0.98	0.11	0.91
Symptoms alone: VD and/or LAP	155	(22)	14	(9)	0.21	0.78	0.94 (0.57–1.49)	1.02	0.09	0.90
Signs alone: VD (excl. candidiasis)	227	(32)	30	(13)	0.45	0.69	1.45 (1.06–1.89)	0.78	0.13	0.92
**Risk scores†, sensitivity minimum 0.7**										
Sociodemographic risk score	327	(47)	50	(15)	0.75	0.56	1.71 (1.42–1.99)	0.45	0.15	0.96
Clinical risk score	372	(53)	54	(15)	0.81	0.50	1.61 (1.37–1.83)	0.39	0.15	0.96
Microscopy risk score	273	(39)	51	(19)	0.76	0.65	2.18 (1.80–2.55)	0.37	0.19	0.96
**Risk scores, sensitivity minimum 0.4**										
Sociodemographic risk score	156	(22)	29	(19)	0.43	0.80	2.17 (1.55–2.91)	0.71	0.19	0.93
Clinical risk score	117	(17)	29	(25)	0.43	0.86	3.13 (2.20–4.30)	0.66	0.25	0.94
Microscopy risk score	116	(17)	29	(25)	0.43	0.86	3.16 (2.22–4.34)	0.66	0.25	0.94

LR+ = positive likelihood ratio; LR- = negative likelihood ratio; PPV = positive predictive value; NPV = negative predictive value; VDS = vaginal discharge syndrome;

LAP = lower abdominal pain; VD = vaginal discharge

* The positive likelihood ratios are calculated with 95% confidence interval.

†Risk factors included in each risk score are described in Table 1

All risk scores suffered from the choice between low sensitivity and low specificity. With a cut-off taken at an acceptable sensitivity of minimum 0.7, the risk score based on sociodemographic factors identifies 50 (75%) and misses 17 (25%) of the 67 cervical infections. Per true case treated, six pregnant women would be prescribed multiple antibiotic regimens unnecessarily. With a cut-off at a sensitivity of 0.4, the sociodemographic risk score identifies only 29 (43%) of the cervical infections. The majority of the cervical infections would remain untreated, and although over-treatment is reduced, four pregnant women would still be unnecessary treated with multiple antibiotics per infected women. The more comprehensive clinical and microscopy risk scores show similar results.

## Discussion

Pregnant women are usually considered to be a low-risk population, but among antenatal care attendees in Botswana, the burden of reproductive tract infections is very high. The prevalence of cervical infections is 10%, and most of the infected women go undetected through normal antenatal care services. Published studies of STIs among pregnant women in other countries in sub-Saharan Africa show similar trends in the prevalence of cervical infections. In our study, 8% have a *C trachomatis *infection, compared to rates between 6% in Tanzania and 13% in Cape Verde [13,22]. Gonorrhea is found in 3% of the women, compared to rates that range from 2% in Gabon to 8% in South Africa [16,21]. Although difficult to measure, it is likely that complications and sequelae due to these infections among pregnant women and their infants in sub-Saharan Africa are substantial.

### Syndromic approach

Elicited symptoms of increased discharge or lower abdominal pain are not predictive of cervical infection in antenatal attendees in Botswana. These symptoms are non-specific; they are common in pregnancy, and their association with cervical infections is even lower among pregnant than among non-pregnant women [23]. The VDS algorithm is used extensively to diagnose cervical infections in antenatal care in Botswana [24], but our results show that this management is no better than random treatment.

To use the genital examination at the first antenatal care visit as a clinical screening tool for cervical infections is also unadvisable. According to the newly revised VDS algorithm in Botswana, symptoms or signs of yellow discharge are risk factors which should lead to treatment for cervical infections. In our study among antenatal care attendees, neither symptoms nor signs of yellow discharge are associated with cervical infection. In pregnancy, symptoms or signs of vaginal discharge in general, yellow discharge, or other specific discharge characteristics should not be used as criteria for treating *C trachomatis *and *N gonorrhoeae*.

Other studies from sub-Saharan Africa conclude that case management with the vaginal discharge syndrome has poor discriminatory ability in the diagnosis of cervicitis [25-27]. Several reviewers [28-30] emphasise that the syndromic approach should not be used as a screening tool for *N gonorrhoeae *and *C trachomatis*. Our study concurs with a substantial body of knowledge indicating that the syndromic approach should be used neither as a case management of symptomatic women nor as a clinical screening tool to identify *C trachomatis *and *N gonorrhoeae *in antenatal care attendees in sub-Saharan Africa.

### Screening strategies

Despite extensive analyses, all computed risk scores were of limited value as screening tools in antenatal care attendees. They had poor discriminative ability, even in the study population in which they were computed and adapted to under optimal conditions. As the syndromic approach, the risk scores also resulted in a large number of undetected cervical infections and substantial over-treatment. Additionally, notification and treatment of sexual partners, an essential element of STI management, is difficult to justify when the majority of the identified women do not have an STI [8]. A substantial improvement of the management of cervical infections in antenatal care in developing countries seems impossible without specific diagnostic tests.

The development of simple, rapid tests for *C trachomatis *and *N gonorrhoeae *has been a high priority since the 1990s [9-11]. Major progress has recently been made, and several tests for *C trachomatis *and *N gonorrhoeae *are now on the market [7,32]. The Sexually Transmitted Diseases Diagnostics Initiative at the WHO has begun a programme to field test and systematically evaluate these simple, affordable rapid tests [32,33]. So far, available tests are found to be specific (>90%), but with a variable sensitivity (25–85%) [34-36]. In Botswana, the health system is relatively well functioning, and this country could well serve as an exploratory site for the use of rapid tests for *C trachomatis *and *N gonorrhoeae*. Through the prevention of mother-to-child transmission of HIV programme, all health posts and clinics have lay workers who perform rapid tests for HIV. Simple tests for cervical infections performed by clinicians or lay workers may prove a feasible contribution to the improvement of diagnosis and the reduction of the disease burden of these conditions in this and similar settings.

Consistent with established knowledge on STI epidemiology [6,31], youth is the single factor most strongly associated with *C trachomatis *and/or *N gonorrhoeae *in our study population. Thus age can be useful as a screening tool in the traditional sense, to minimise the number of standard diagnostic tests by identifying people with a higher-than-average prevalence of infection [29]. If it were decided in Botswana to introduce screening for cervical infections with rapid tests in the antenatal care, selective screening of younger women should be considered.

## Conclusion

Although the vaginal discharge syndrome does not discriminate between infected and uninfected women, the algorithm is in extensive use to diagnose and treat *C trachomatis *and *N gonorrhoeae *among antenatal care attendees in Botswana. Unfortunately, risk scores do not appear to improve the management of cervical infections in pregnancy substantially. To diagnose and treat asymptomatic cervical infections, and to reduce the massive overtreatment in the syndromic management, specific diagnostic tests are necessary. Screening for cervical infections in pregnant women is an essential public health measure, and rapid tests will hopefully be available in developing countries within a few years. In the temporary absence of such tests, health authorities in sub-Saharan Africa should consider reallocating their resources to other STI measures rather than diagnosing and treating gonorrhoea and chlamydia inadequately in antenatal care.

## Abbreviations

STI, sexually transmitted infection; VDS, vaginal discharge syndrome; PMN/HPF, polymorphonuclear leukocutes per high power field; OR, odds ratio; ROC, receiver operating characteristics; LR+ and LR-, positive and negative likelihood ratios; PPV and NPV, positive and negative predictive value; CI, confidence interval.

## Competing interests

The author(s) declare that they have no competing interests.

## Authors' contributions

MR contributed to the study design, was responsible for data collection and data analysis, and drafted the manuscript. MRa contributed to the study design and to formal and organisational aspects of the study. MV contributed to the study design, and led and performed the majority of the laboratory work. JS and PH supervised the study. All authors read and approved the final manuscript.

## Pre-publication history

The pre-publication history for this paper can be accessed here:


